# Canine mammary cancer tumour behaviour and patient survival time are associated with collagen fibre characteristics

**DOI:** 10.1038/s41598-021-85104-w

**Published:** 2021-03-11

**Authors:** Ana P. V. Garcia, Luana A. Reis, Fernanda C. Nunes, Francis G. J. Longford, Jeremy G. Frey, Ana M. de Paula, Geovanni D. Cassali

**Affiliations:** 1 Laboratório de Patologia Comparada, Instituto de Ciências Biológicas, Universidade Federal de Minas Gerais, Belo Horizonte, MG 31270-901 Brazil; 2 Departamento de Física, Instituto de Ciências Exatas, Universidade Federal de Minas Gerais, Belo Horizonte, MG 31270-901 Brazil; 3 University of Southampton, Southampton, SO17 1BJ UK

**Keywords:** Imaging, Multiphoton microscopy, Cancer, Cancer imaging

## Abstract

Precise diagnosis and prognosis are key in prevention and reduction of morbidity and mortality in all types of cancers. Here we show that changes in the collagen fibres in the main histological subtypes of canine mammary gland carcinomas are directly associated with the tumour behaviour and the animal survival time and could become a useful tool in helping with diagnosis. Imaging by second harmonic generation and multiphoton excited fluorescence microscopy were performed to evaluate the collagen and cellular segment parameters in cancer biopsies. We present a retrospective study of 45 cases of canine mammary cancer analysing 836 biopsies regions including normal mammary gland tissue, benign mixed tumours, carcinoma in mixed tumour, carcinosarcoma, micropapillary carcinoma and solid carcinoma. The image analyses and the comparison between the tumour types allowed to assess the collagen fibre changes during tumour progression. We demonstrate that the collagen parameters correlate with the clinical and pathological data, the results show that in neoplastic tissues, the collagen fibres are more aligned and shorter as compared to the normal tissues. There is a clear association of the mean fibre length with the dogs survival times, the carcinomas presenting shorter collagen fibres indicate a worse survival rate.

## Introduction

Breast cancer is the most common cancer among women—with the exception of non-melanoma skin cancer—corresponding to about 25% of new cases each year worldwide. In Brazil, this rate is even higher, reaching 29.7%, according to the National Cancer Institute^1^. Thus it is of great importance for public health that motivates studies on prevention and early diagnosis, in the search for the reduction of morbidity and mortality related to this neoplasm^2^. The subject is also of great interest in Veterinary Medicine as about 50% of the tumours in female dogs are malignant mammary neoplasms^3^, largely a reflection of late diagnosis, which compromises treatment and reduces the survival rate of the animals^4–6^. In addition, mammaryneoplasms in female dogs and women show epidemiological, clinical, biological and genetic similarities, which makes it possible to use the female dog as a comparative model^6–13^.

It is known that breast neoplasms stimulate the degradation of components of the extracellular matrix. Collagen, specifically, undergoes significant structural changes in the presence of malignancy that plays an important role in modulating the behaviour of breast cancers^14–30^. The changes in the collagen structural architecture have been used also to successfully detect malignancies in the canine mammary gland^31^. In addition, collagen plays a significant role in the emergence of metastases, with a large amount of studies of cancer prognosis and therapies^32–35^. Recent studies indicate that there must be a balance between the magnitude of the traction forces and the adhesion force of the cell to the extracellular matrix to achieve an ideal process of cell migration^36–40^. In a multicellular system, the tensile forces generated by individual cells can give rise to an evolving force network (supported by the fibres of the extracellular matrix), that actively pull the individual cells in order to move them in the extracellular matrix and this force can be detected by distant cells^40^. The mechanical coupling between the cells can influence the migration of individual cells, which could alter the structure and properties of the extracellular matrix and, therefore, the tensile strength network. This feedback loop between the strength of the extracellular matrix network and cell migration may be responsible for a wide range of collective migratory behaviour^36–40^.

Therefore, understanding the structure and functional properties of the collagen fibres is essential for understanding the tumour behaviour in different types of neoplasms. The collagen is a non-centrosymmetric fibre that efficiently generates second harmonic signals, thus images obtained by nonlinear microscopy from second harmonic generation (SHG) have been shown to be a useful method to study these tissue changes^32,41^. The SHG microscopy allows to obtain data regarding the morphology of the extracellular matrix, including the organization, shape and quantification of the collagen fibres that make up this matrix, characterizing the changes that occur in the fibres during tumour progression that facilitates the extravasation and migration of tumour cells in many types of cancers^14,16,18,22,23,31,37,42,43^. Recent polarization resolved SHG results have shown details of the collagen microscopic structure modifications that can discriminate the histological grades of breast cancer^30^. And the details of the macroscopic collagen organization obtained by SHG microscopy image analysis have been shown to correlate with the survival of luminal^28^ and triple negative breast cancer patients^44^. In addition, it has been shown that third harmonic generation microscopy allows to obtain details of the tumour cells with potential for differentiating malignant from benign breast tissue^45^.

Most of the previous studies measured the collagen properties at the tumour borders, however recent studies have shown that the intra tumoral collagen properties are important for the differentiation of invasive tumours^29,35^. Here we present results for multiphoton microscopy, by simultaneous two-photon excited fluorescence (TPEF) and SHG imaging, together with an image analysis procedure that provided a range of collagen and cellular segment parameters of intra-tumour tissue. The images were performed on archive standard hematoxylin and eosin (H&E) stained histological slides from 45 patients and allowed a correlation with the animal survival time. A large number of intra-tumoral representative areas of each patient were imaged (836 biopsies regions) to cover the heterogeneity of the tumours. We implemented a comprehensive and robust image analysis methodology to extract the collagen fibre network and to quantify the properties of the collagen fibres and the cellular segments in the images. A software package was developed to perform an automated image segmentation into collagen and cellular regions^46^ and to extract their parameters (available on GitHub^47^). The measured parameters include the organization of the fibres, the number of fibres, the mean fibre length, the shape of the cellular segments and the proportion of the image area covered by fibres or cellular segments. We demonstrate that the obtained parameters allows a good discrimination of the main histological types of canine mammary neoplasms. In addition, the results show that the changes in the collagen fibre length directly correlate with the tumoral behaviour and the animal survival time.

## Results and discussion

### Details of the samples studied

The details of the histological subtypes analysed: the age of the patients, clinical stage, histological grade, molecular subtype, cell proliferation rate and survival times are shown in Table 1. The mean age of all the patients was of 10 years. The mean age for each histological subtype is presented in Table 1with the standard deviation in brackets. Among the 45 cases of mammary neoplasm evaluated, 36% were carcinoma in mixed tumour (CMT; $n = 16$), 26% solid carcinoma (SC; $n = 12$), 16% micropapillary carcinoma (MC; $n = 7$), 13% benign mixed tumour (BMT; $n = 6$) and 9% carcinosarcoma (CS; $n = 4$). In relation to the clinical staging, 44% of the carcinomas were classified with staging IV ($n = 20$), 22% with staging I ($n = 10$), 18% with staging III ($n = 8$), 9% with staging V ($n = 4$) and 7% with staging II ($n = 3$). For the histological grade 36% of the cases were classified with histological grade II ($n = 16$), 33% with histological grade III ($n = 15$) and 18% with histological grade I ($n = 8$). Regarding the molecular subtype, 25% of the cases were classified as luminal A ($n = 10$), 67% as luminal B ($n = 26$) and 8% as triple negative ($n = 3$). Among the analysed cases, 60% had low cell proliferation rate (Ki67 $\le 20$%; $n = 27$) and 40% of the cases had a high cell proliferation rate $({\hbox {Ki67}}> 20$%; $n = 18$). The mean survival time of the patients are presented in Table 1 with the standard deviation in brackets. The patients diagnosed with BMT were all alive until the last contact with the person responsible for the dog, that was greater than 658 days. Thus this value was not included in the statistical analyses.

**Table 1:** Clinicopathological features of canine mammary neoplasms.

Histolo-gical type	n(%)	Age (year)	Staging					Histological grades				Molecular subtype				Survival (days)
			I	II	III	IV	V	I	II	III	NA	HR+/Ki67 $\le \, 20$%	HR+/Ki67 $>\,20$%	HR- /HER2-	NA	
BMT	6 (13%)	9.0 (2.2)	4	2	0	0	0	0	0	0	6	0	0	0	6	658
CMT	16 (36%)	10.6 (2.8)	4	1	4	7	0	8	8	0	0	8	7	1	0	687 (375)
MC	7 (16%)	11.0 (3.4)	0	0	0	5	2	0	4	3	0	1	6	0	0	152 (110)
CS	4 (9%)	9.3 (4.2)	0	0	3	0	1	0	0	4	0	0	3	1	0	165 (149)
SC	12 (26%)	10.2 (4.0)	2	0	1	8	1	0	4	8	0	1	10	1	0	267 (220)
Total	45 (100%)		10	3	8	20	4	8	16	15	6	10	26	3	6	

For the age and the survival, the data are the mean values with the standard deviation in brackets.

*BMT* benign mixed tumour, *CMT* carcinoma in mixed tumour, *MC* micropapillar carcinoma, *CS* carcinosarcoma, *SC* solid carcinoma.

### Images and extracted parameters

**Figure 1:**
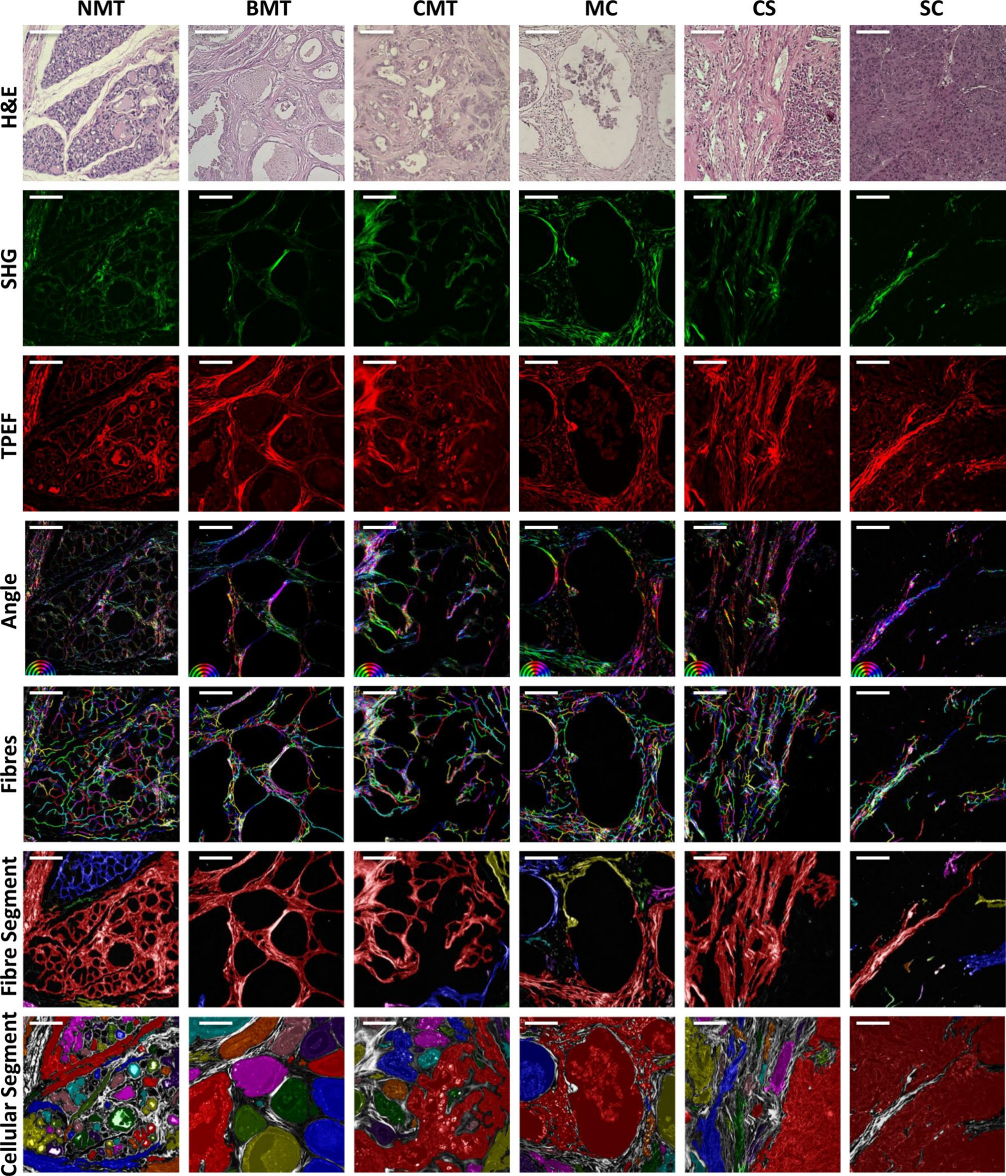
Acquired images: the optical microscopy H&E (first row), SHG (second row), TPEF (third row) and the images extracted by the software: a colour map image with the angle distribution of the collagen fibre orientation in the tissue (fourth row, angle colour map at the bottom left hand side), the extracted collagen fibres with each fiber in a random colour (fifth row), the fibre segment (sixth row) and the cellular segments (seventh row) for the histological types in the columns NMT, BMT, CMT, MC, CS, SC. Each connected fibre and cellular segment is presented in a random colour. The scale bar is 100 $\upmu$m.

Figure 1 presents representative images of the histological mammary sections studied. The histological types in the columns are indicated as NMT, BMT, CMT, MC, CS and SC for the normal mammary gland, benign mixed tumour, carcinoma in mixed tumour, micropapillary carcinoma, carcinosarcoma and solid carcinoma, respectively. The rows one to three show the measured images and the extracted fibre and cellular segmented images obtained from the image analysis methodology are in rows four to seven. The H&E bright field microscopy optical images are in the first row, the SHG images in the second row and the TPEF images in the third row. The TPEF signal is the fluorescence emission of the eosin dye. The SHG and TPEF are false colour images of the measured intensity maps normalized to highlight all the features. The fourth row shows a colour map image with the angle distribution of the collagen fibre orientation in the tissue (the angle colour map is at the image bottom left hand side). Note that the NMT image show a more isotropic distribution of angles, whereas the solid carcinoma image shows almost all fibres oriented in one direction indicated by the purple colour. In the fifth row are the images of the extracted network of collagen fibres with the individual fibres in random colours. The sixth row shows the connected collagen fibre segments, each segment in a different colour. And in the seventh row is the cellular segments, where each connected cell-cluster is in a different colour. The fibre segment images are shown superimposed on the SHG images in grayscale and the cellular segment images are presented superimposed on the TPEF images in grayscale with each connected cellular segment in a different colour. The details of the image measurements and analysis are described in the “Materials and methods” section.

The normal mammary gland, first column, presents acini and ducts consisting of luminal epithelial cells lined by myoepithelial cells that are separated from the surrounding connective tissue by the basement membrane^48,49^. The collagen fibres, abundant in the surrounding connective tissue, are arranged in different directions throughout the mammary tissue. The benign mixed tumour (BMT), second column, are characterized by benign proliferation of cells morphologically similar to the epithelial (luminal or myoepithelial cells) and mesenchymal components that produce cartilage and/or bone and/or adipose tissue, possibly in combination with fibrous tissue^50^. In these neoplasms, it is still possible to observe an abundance of collagen fibres in the surrounding connective tissue, however the fibres are more organized in relation to the NMT. The carcinomas in mixed tumour (CMT), third column, exhibit a complex histological pattern, as they have components of epithelial and mesenchymal origin. Malignant epithelial cells exhibit an infiltrative growth, which can be identified by the loss of continuity of the basal/myoepithelial layer associated with clusters of tumour cells that penetrate the stroma. The occurrence of non-invasive proliferation (in situ) can also be observed. The differentiation between in situ and invasive components in carcinoma in mixed tumours is possible due to the presence of stromal invasion and microinvasion. The areas of invasion are characterized by the presence of clusters of infiltrative tumour epithelial cells in the regions of periductal stroma close to the components of the carcinoma. Carcinoma in situ with microinvasion areas is defined when the continuity of the basement membrane is lost, within 1 mm, and epithelial cells are present^51^. In these carcinomas, it is possible to observe a greater organization of collagen fibres that make up the surrounding connective tissue in relation to the NMT and BMT. It is possible to observe the alignment of the collagen fibres in the connective tissue that surrounds the neoplastic growth. The micropapillary carcinomas (MC), fourth column, exhibit well-defined cystic spaces similar to the lymphatic vessels diffusely distributed throughout the mammary gland. Within these cystic spaces there are clusters of epithelial cells with a micropapillary pattern called “moruliform”, with abundant eosinophilic cytoplasm, evident nuclear pleomorphism and prominent nucleoli^51^. In these carcinomas, it is also possible to notice the orientation of the collagen fibres in a preferred direction, indicating greater organization of the surrounding connective tissue. The histological characteristics of the carcinosarcomas (CS), fifth column, are extremely variable and were previously described as mixed malignant tumours of the mammary gland. They are composed of malignant epithelial and mesenchymal cells^51^ and show an abundance of connective tissue. In the solid carcinoma (SC), sixth column, there is a proliferation of epithelial cells organized in a solid arrangement, with the formation of cords, sheets or agglomerates. The tumour cells are undifferentiated, exhibit small hyperchromatic nuclei and the mitotic index is high. The amount of stroma can vary from small to moderate and areas of necrosis are frequently observed^51^. As in the other carcinomas, in the solid carcinoma it is also possible to observe greater organization of the collagen fibres of the surrounding connective tissue in relation to NMT and BMT.

Our image analysis procedure and the extracted fibre and cellular segments (see details on the “Materials and methods” section) allowed the quantification of many of the image visual characteristics described above. The colour map images of the angle distribution of the collagen fibre orientation in the tissue (fourth row) show that the carcinomas with a worse prognosis present more aligned fibres. Also the images of the collagen fibre networks (fifth row) show the decrease of the number of fibres for the carcinomas with worse prognosis. And the comparison of the fibre and cellular segments in the sixth and seventh rows shows the changes of the areas covered by fibres and cells.

**Figure 2:**
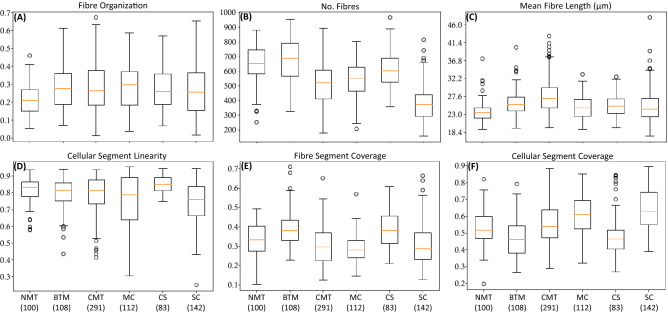
Boxplot graphics showing the calculated parameters for the fibre and cellular segments: the fibre organization, number of fibres, the mean fibre length, the cellular segment linearity and the image area covered by the fibre and cellular segments for all the tumour types studied. The numbers below each histological type indicate the number of acquired images. The centre lines show the medians, the box limits indicate the 25th and 75th percentiles, the whiskers extend 1.5 times the interquartile range from the 25th and 75th percentiles and the outliers are represented by dots.

We will discuss some of the measured parameters that allow a good differentiation between the histological type. The collagen fibre organization parameter (fibre organization), the number of collagen fibres, the mean fibre length, the linearity of the cellular segments and the image area covered by fibre (fibre segment coverage) and by cellular regions (cellular segment coverage) for all the histological types are shown in Fig. 2. The statistical correlations between the histological types are shown in the Table 2.

The collagen fibre organization parameter is a measurement of the degree of alignment of the collagen fibres, that ranges from zero to one for random organized fibres and for well oriented fibres all aligned along a specific direction in the tissue, respectively. The results in Fig. 2A show that in all histological types the collagen fibres are more aligned as compared to the NMT and it provides a clear separation between normal and neoplastic tissue. These findings corroborate with others described in the literature^14,15,17,19–21,24–29,31,52,53^.

The number of fibres and fibre length are two important parameters to assess the changes of the collagen in neoplastic tissues. The number of fibres, Fig. 2B shows that the amount of collagen decreases in the neoplastic tissues within the tumour regions as compared to the NMT except for the BMT, due to the large amount of extracellular matrix characteristic of this histological type^9,54,55^. However, the fibres are longer in the neoplastic tissue, Fig. 2C. Nonetheless, between the tumour types the collagen fibres show shorter length in the MC, CS and SC as compared to the BMT and CMT. These results lead to the hypothesis that within the tumour types, the less aggressive mammary carcinomas present longer collagen fibres than the more aggressive ones. These two parameters show statistically significance between most of the histological types, Table 2.

The cellular segment linearity is presented in Fig. 2D. This is a parameter that evaluates the shape of the extracted segments of cellular regions and it measures the elongation of the segments as compared to a circle. It may be expected that normal tissue types will contain cellular regions that display more regular, circular appearance, whereas the value of the linearity metric for this type suggests that they are more elongated. An explanation for this may be seen in Fig. 1; although visually the cellular regions of the normal type image (first column) are relatively circular, the colored cellular segments identified by the analysis software are highly folded, due to the presence of the collagen structures within the intra tumoral regions that are detected by the SHG. This folding leads to a much greater value for the segment perimeter than would be expected by eye. Note that the MC and SC show the more circular shapes, in these tissue types, the cellular regions display less collagen within the cell mass and so the cellular segments are not folded. Thus, this parameter allows a good discrimination of the cancer types. The *p*-values show statistically significance between most of the histological types, Table 2.

For the parameters, fibre segment coverage and cellular segment coverage, shown in Fig. 2E, F and Table 2, it is observed that the BMT and CS show a higher amount of collagen and less cellular segments, corroborating with studies that used other methodologies^9,54,55^. In addition, the results show a larger area covered by cellular segments in the SC as compared to other histological types in agreement with the characteristic of solid carcinomas that show an expansive growth in highly cellularized nests^13^. For the comparisons between the histological types, the BMT presented a larger area covered by fibres in comparison to the samples of the other histological subtypes^9,54^. These parameters also show statistically significance between most of the histological types.

**Table 2:** Statistical comparisons between the diagnostic groups.

	NMT $\times$ BMT	NMT $\times$ CMT	NMT $\times$ MC	NMT $\times$ CS	NMT $\times$ SC	BMT $\times$ CMT	BMT $\times$ MC	BMT $\times$ CS	BMT $\times$ SC	CMT $\times$ MC	CMT $\times$ CS	CMT $\times$ SC	MC $\times$ CS	MC $\times$ SC	CS $\times$ SC
Fibre organization	***	***	***	***	**										
No. fibres		***	***		***	***	***	**	***		***	***	***	***	***
Mean fibre length	***	***	**	***	**	***	**			***	***	***			
Cellular segment linearity			***	***	***		**	***	***	***	***	***	***		***
Fibre segment coverage	***	*	***	***		***	***		***		***		***	*	***
Cellular segment coverage	***	*	***	*	***	***	***		***	**	***	***	***		***

*NMT* normal mammary tissue, *BMT* benign mixed tumour, *CMT* carcinoma in mixed tumour, *MC* micropapillar carcinoma, *CS* carcinosarcoma, *SC* solid carcinoma. The *p* values were obtained by one-way ANOVA and Tukey’s HSD test.

*p* value significance: $p < 0.05$ are denoted as *, $p < 0.01$ are denoted as ** and $p <0.001$ are denoted as ***. Empty table cells denote not significant *p*.

### Changes in the collagen parameters and tumour progression

**Figure 3:**
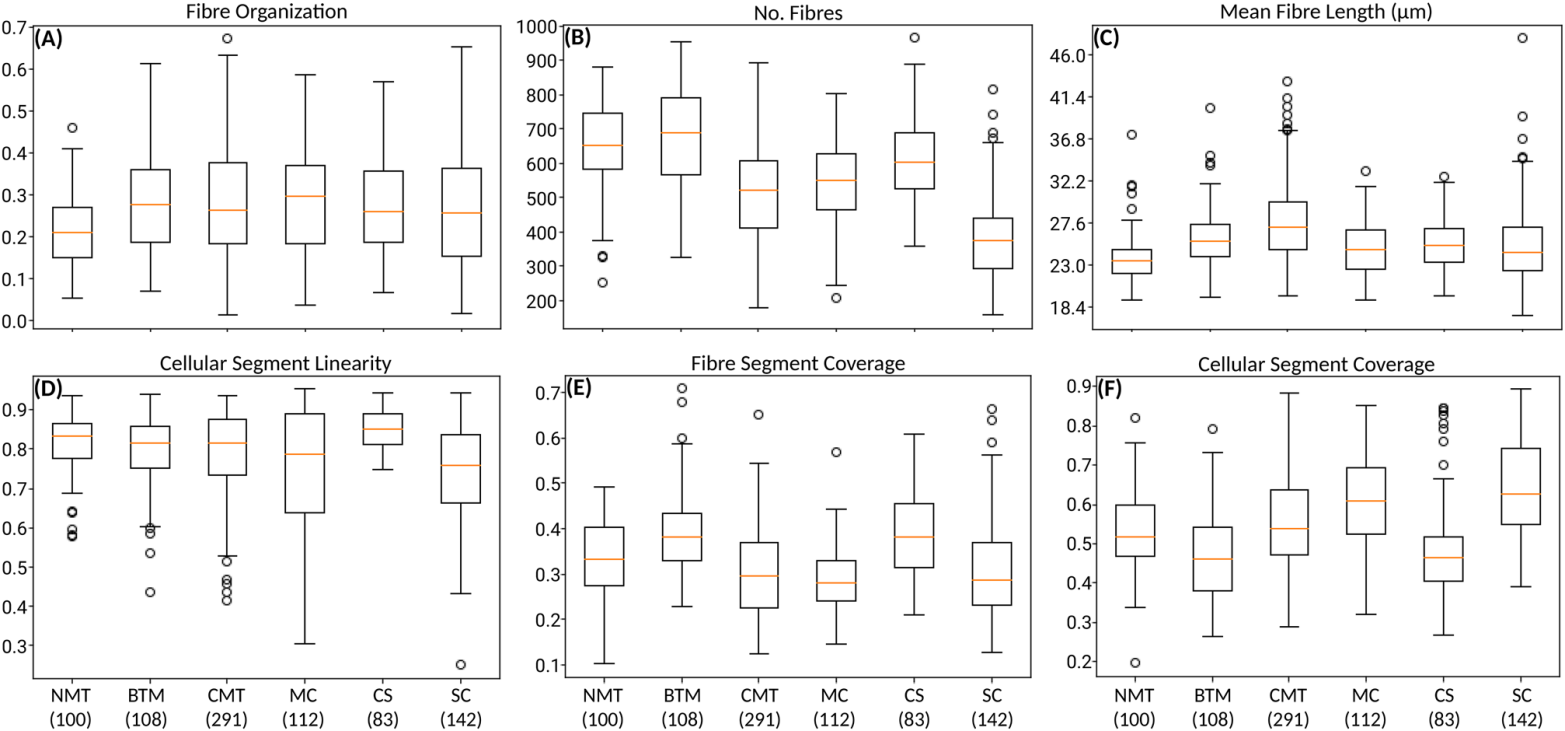
Boxplot graphics showing the calculated parameters for the fibre and cell segments: the fibre organization, number of fibres, the mean fibre length, the cellular segment linearity, the fibre segment coverage and the cellular segment coverage for the NMT, CMTis and CMTi regions of the CMT. The centre lines show the medians, the box limits indicate the 25th and 75th percentiles, the whiskers extend 1.5 times the interquartile range from the 25th and 75th percentiles and the outliers are represented by dots.

Here we show the measured parameters for the CMT to discuss the collagen changes as the tumour progress. The results obtained for the fibre organization, number of fibres, collagen fibre length, cellular segment linearity, fibre segment coverage and cellular segment coverage are presented in Fig. 3. It is shown the comparisons between the NMT and the benign areas (CMTb), the malignant in situ (CMTis) and the malignant invasive regions (CMTi) of the CMT. The statistical data are shown in Table 3. The benign areas are not so common, thus only 24 regions were imaged.

For the fibre organization parameter, Fig. 3A, the results show that the collagen fibres are more aligned in the CMTis and CMTi regions of the CMT in comparison to the NMT and the CMTb, but there is no clear separation between the NMT and the CMTb. However, it is possible to say that during tumour progression the collagen fibres become more aligned as the neoplasia develops. It is well known that cellular migration involves integration into a complex microenvironment, which can be a physiological or pathological process, including regeneration tissues, immune response and tumor progression^12,36–40,56–58^. In addition to well-established chemotaxis, the microstructure and physical properties of the extracellular matrix have a significant influence on cell migration via durotaxis^12,56–58^. This process can be explained as a unidirectional cell migration mechanism in which a cell responds to an extracellular gradient of rigidity, which is important in the process of cell migration and invasion in tumour progression. During durotactic migration, there is usually cellular movement towards regions of increasing stiffness on increasingly rigid substrates^36–40^. Thus, the changes in the alignment and the amount of collagen in the tumor microenvironment may be capable of facilitating the migration and invasion of neoplastic cells in other locations.

For the number of fibres, the data presented in Fig. 3B and Table 3 show that the CMTb presents a significant smaller amount of fibres than the NMT. The number fibres keeps decreasing for the CMTis areas but increases again for the CMTi areas. However, the mean fibre length shows the opposite trend, Fig. 3C, the collagen fibres are longer in CMTb, CMTis and CMTi than in the NMT. Therefore, it is possible to say that during tumour progression the collagen fibre length increases as the neoplasia develops. However, in the malignant invasive regions the fibres decrease again in comparison to the malignant in situ regions. These two parameters show statistically significance between most of the histological types and are good indicators of the tumour progression, Table 3. Several studies describe that neoplastic cells influence and are influenced by the extracellular matrix and the tumour microenvironment during tumour progression, in order to invade the underlying tissues^12,36–40,56–58^. Thus, as discussed earlier, we believe that for the in situ growth profile the collagen fibres are larger and more stretched, limiting the neoplastic growth. Nonetheless, when the cells break the basal membrane and express an invasive profile, the fibres become more fragmented and shorter as compared to the previous state.

The linearity of the cellular regions, Fig. 3D show more circular shapes for the CMTb areas as compared to both the NMT and the CMTis and CMTi areas. Thus this parameter can separate the CMTb from the NMT. For the parameters fibre coverage and cellular segment coverage, the results presented in Fig. 3E, F and Table 3 show that the benign areas of the CMT have a smaller fibre coverage area as compared to NMT and the malignant in situ and invasive areas. Thus, the benign areas show larger cellular segment coverage in comparison to the NMT and the malignant in situ and invasive areas of CTM. These results confirm that at the later stages of tumour progression, the cell proliferation is more pronounced in relation to the collagen deposition^12,36–40,56–58^.

**Table 3:** Statistical results for the tumour progression.

	NMT $\times$ CMTb	NMT $\times$ CMTis	NMT $\times$ CMTi	CMTb $\times$ CMTis	CMTb $\times$ CMTi	CMTis $\times$ CMTi
Fibre organization		***	***			
No. fibres	**	***	***			
Mean fibre length	***	***	***			*
Cellular segment linearity	***	**			***	**
Fibre segment coverage		***				***
Cellular segment coverage	**	***				**

*p* value significance: $p < 0.05$ are denoted as *, $p < 0.01$ are denoted as ** and $p <0.001$ are denoted as ***. Empty table cells denote not significant *p* *NMT* normal mammary gland, *CMTb* benign areas of carcinoma in mixed tumour, *CMTis* in situ areas of carcinoma in mixed tumour, *CMTi* invasive areas of carcinoma in mixed tumour. The *p* values were obtained by one-way ANOVA and Tukey’s HSD test.

### Carcinomas with worse prognosis present shorter collagen fibres

**Figure 4:**
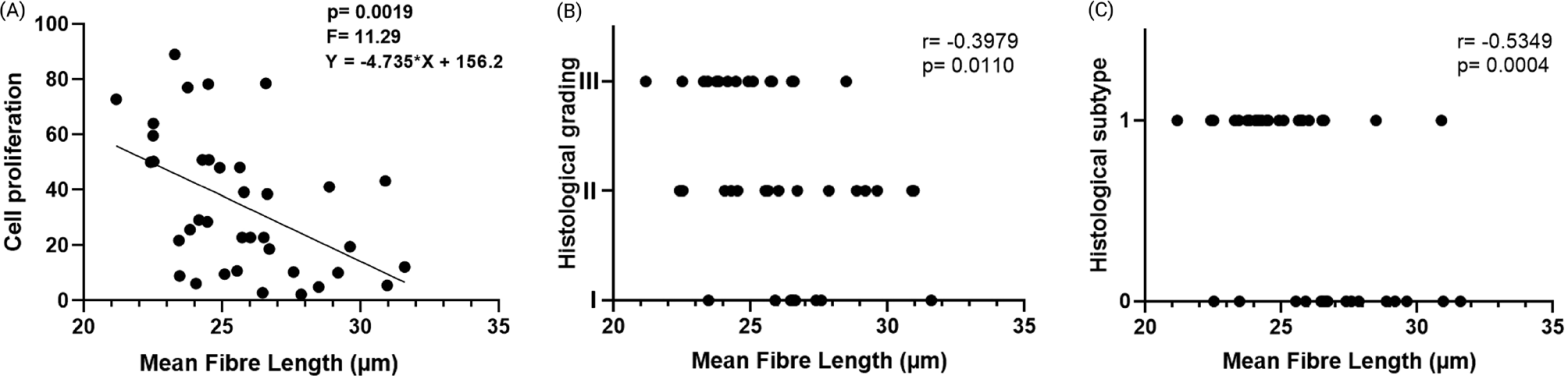
(**A**) Linear regression for the fibre length and the cell proliferation; (**B**) Spearman’s correlation for the fibre length and the histological grades I, II and III; (**C**) Spearman’s correlation for the fibre length and the histological subtype, where the subtype is presented as 0 for carcinomas with good prognosis (CMT) and 1 for carcinomas with worse prognosis CS, MC and SC).

Figure 4 shows the analyses of the collagen fibre length of all the malignant histological types studied (CMT, CS, MC and SC) with the cell proliferation, histological grading and histological subtype. The linear regression for the fibre length and the cell proliferation is presented in Fig. 4A. The Spearman’s correlations for the fibre length and the histological grades I, II and III are in Fig. 4B, and the Spearman’s correlation for the fibre length and the histological subtypes are in Fig. 4B. The histological subtypes are presented as 0 for carcinomas with good prognosis (CMT) and 1 for carcinomas with worse prognosis (CS, MC and SC). All the correlations show that the carcinomas with worse prognosis present shorter collagen fibres. Correlations of the mean collagen fibre length with other clinical and pathological data were performed, such as patient age, clinical staging, tumor size, lymph node status, ER, PR, HER2, but they did not show statistical correlations.

**Figure 5:**
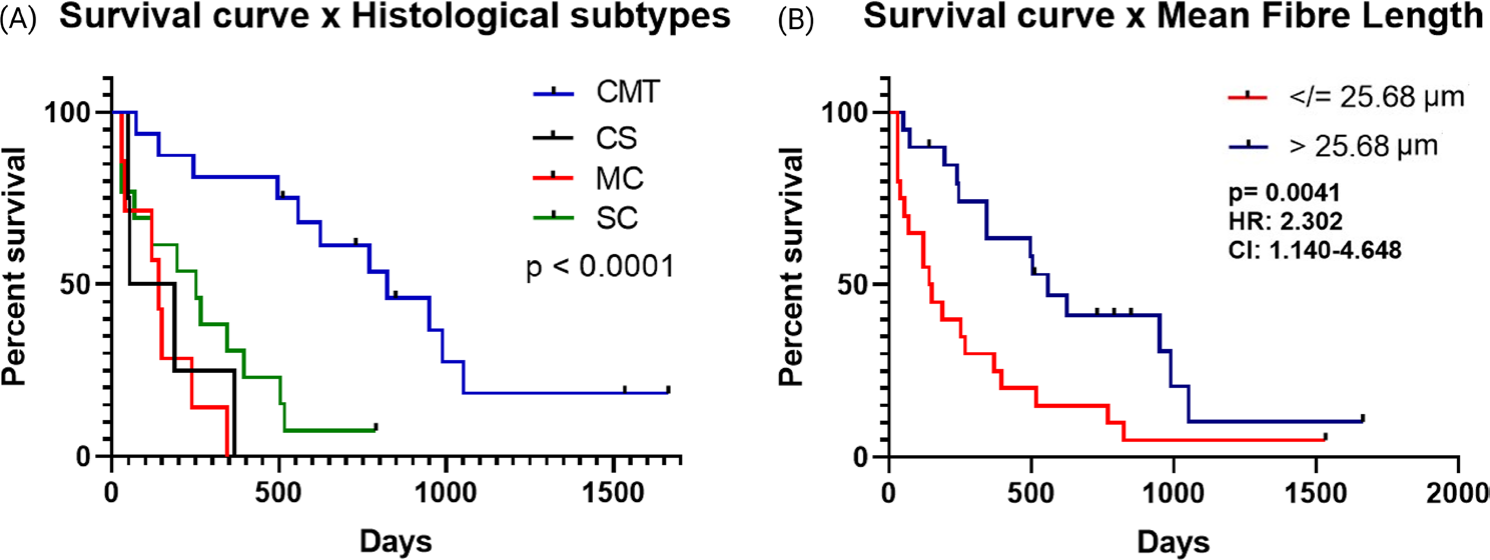
(**A**) Survival curves for dogs presenting the histological subtypes, *CMT* carcinoma in mixed tumour, *CS* carcinosarcoma, *MC* micropapillary carcinoma, *SC* solid carcinoma. (**B**) Survival curves separating the cases by the collagen mean fibre length, the red line for fibre length $\le 25.68\,\upmu$m and the blue line for fibre length $> 25.68\,\upmu$m.

In Fig. 5 we present the survival curves for the dogs diagnosed with each histological subtypes, Fig. 5A, and with the dogs grouped by the mean fibre length disregarding the histological type, Fig. 5B. Figure 5A shows that the dogs diagnosed with CS reached the median survival time in 54 days, the ones diagnosed with MC in 140 days, the SC in 252 days and the CMT in 824 days. The COX regression analysis was performed using the CMT as a reference for a carcinoma with a better prognosis. However, due to the low number of cases of the other histological types (CS, MC and SC) they were grouped into a single group considering that they all have a poor prognosis in relation to CMT, as demonstrated by previous studies^13,55,59^. Thus only one *p* value is shown. Correlating this information with the data presented in Fig. 2C, it is possible to note that the carcinomas with worse prognosis present shorter collagen fibres in comparison to the CMT. From this analysis, a cut-off point was established to determine whether the collagen fibre length could be used as a clinical complementary parameter for the diagnosis of mammary cancer. To obtain a cut-off point we considered all the histological subtypes together. The median fibre length of 25.68 $\upmu$m was obtained and considered as the cut-off. Then the dogs were stratified into two groups defined by this median cut-off point, disregarding histological subtype, histological grading and cell proliferation rate. The numbers 0 and 1 were assigned to the distribution of cancer-specific survivals: 0 was assigned to dogs diagnosed with carcinomas presenting collagen fibre length smaller than the cut-off and number 1 was assigned to dogs diagnosed with carcinomas presenting collagen fibre with length larger than the cut-off. In this way the prognostic and predictive factors did not interfere in the correlations established between the mean fibre length and the survival of the animals in the study. The results are presented in Fig. 5B, the dogs diagnosed with carcinomas that present short collagen fibres (number 0) reach the median survival time on the 150th day and dogs diagnosed with carcinomas with long fibres (number 1) reach the median survival time on the 511th day (HR = 2.302, *p* = 0.0041, [CI] = 1.140–4.648). Therefore, the results clearly indicate that the more aggressive carcinomas, with unfavorable prognosis, present shorter collagen fibres as compared to less aggressive ones. It should be pointed out that the acquired images were for regions within the tumour mass. Previous results have shown an opposite trend for the fibres at the tumour border^31^. These results corroborate the previously presented hypothesis that carcinomas with an invasive growth profile and with a tendency to metastasize tend to have shorter fibres in comparison to carcinomas with an in situ and local growth profile^37^.

Nonetheless, in a multivariate analysis, the mean fibre length is not included in the final model as in the univariate risk analysis it presented $p=0.06$. On a multivariate model with the variables: histological subtype, histological grade, clinical stage, estrogen receptor, progesterone receptor, HER-2, molecular subtype, cell proliferation index, clinical stage and mean fibre length only the histological subtype and clinical stage variables were included in the final model. An increased risk of death was observed in dogs diagnosed with carcinomas with stage IV and V (HR = 3.74, $p < 0.010$, confidence interval [CI] = 1.36–10.27) in comparison to the dogs diagnosed with tumour stages I–III and in dogs diagnosed with more aggressive histological subtypes (HR = 11.35, $p < 0.0001$, confidence interval [CI] = 3.14–41.00).

## Conclusion

In conclusion, the biopsy evaluation by the nonlinear microscopy technique and the new image analysis procedure allowed to demonstrate differences in the organization, number and length of the collagen fibres between neoplasms and normal mammary gland during tumour progression. In addition we demonstrated that, among the subtypes of mammary carcinomas, the more aggressive carcinomas present shorter collagen fibres in comparison with the less aggressive ones that directly correlates with the dogs survival times. These imaging and analyses could be useful tools to help towards more precise cancer diagnostics.

## Materials and methods

### Biopsies: case selection

We present a retrospective study of 45 cases of canine mammary cancer and 12 samples of healthy mammary gland regions as control. We included the samples that contained the information on clinical staging, histological graduation, molecular subtype and cell proliferation. The samples were obtained from the Laboratory of Comparative Pathology at Federal University of Minas Gerais (UFMG), Brazil. The materials used for the imaging were the standard histological slides stained with H&E, from fragments of neoplasms fixed in formalin and embedded in paraffin. The tissues were obtained from biopsies of simple mastectomy, regional mastectomy, unilateral or bilateral radical mastectomy depending on the tumour size, clinical staging, lymphatic drainage and tumour location. The selected samples were collected from the years 2009 to 2019 and they were classified according to the criteria described in the Consensus for the Diagnosis, Prognosis and Treatment of Canine Mammary Tumours^51^, including the types: 12 NMT, 06 BMT, 16 CMT, 07 MC, 04 CS and 12 SC. All cases were reviewed in sections stained with H&E by an experienced pathologist specialized in canine mammary gland cancer. Clinical staging was performed based on tumour size (T), neoplastic involvement of regional lymph nodes (N) and presence of distant metastases (M) according to the TNM system established by the World Health Organization (WHO), modified by Owen, for canine mammary tumours^59^. The histological grade was established according to the Nottingham system. For the correlations with the survival data, the patients included were the ones treated with surgery only. The score 1 was used for those who died due to mammary carcinoma and 0 for the ones alive or those who died due to other causes.

The biopsy microscopy slides used were of the routine histopathological diagnosis of spontaneous occurring tumours of female dogs obtained at the Veterinary Hospital of UFMG.

### Ethical approval

The study was performed in view of the fundamental ethical principles of law No. 11.794, of October 8, 2008 and of decree No. 6.899 of July, 2009, and with the rules issued by the National Council for the Control of Animal Experimentation (CONCEA). It was approved by the “Ethics Committee on the Use of Animals” at UFMG, under No. 251/2018.

### Second harmonic generation and two-photon excited fluorescence imaging

The SHG and TPEF imaging system is a home built setup using an Olympus FV300 confocal scanning laser unit attached to an upright BX61-WI microscope^43^. We used a 140 fs Ti-Sapphire oscillator (Coherent Chameleon) with 80 MHz repetition rate tuned to the wavelength of 800 nm. The laser energy per pulse at the sample position is about 0.1 nJ, average power of 7 mW, fluence of $7 \times 10^{-4}$ J/cm$^2$. The laser beam passes through the scanning mirrors and through a dichroic mirror and is focused on the sample at normal incidence by a 20$\times$ objective lens (N.A. 0.90). The laser is circularly polarized at the sample position, that is achieved using a half-wave plate and a quarter waveplate in the laser path before it enters the confocal microscope^60^. The SHG backscattered signal is collected by the same objective and directed by a polarization-insensitive dichroic mirror (Semrock FF665-Di02) to the detector (a photomultiplier tube, PMT). A thin band pass filter (20 nm bandwidth, Chroma HQ400/20m-2p) centred at the SH wavelength (400 nm) a blocking edge filter (Semrock FF01-680/SP-25) are used to completely remove the laser scattered light.

For the TPEF imaging the dichroic mirror is moved out of the beam and the backscattered signal follows the descanned path and is measured by the internal PMT of the confocal. The signal is filtered by a band pass filter in the range 560–600 nm and the blocking edge filter (Semrock FF01-680/SP-25). The TPEF signal is the fluorescence emission of the eosin dye in the H&E stained tissues. We collected SHG and TPEF images at the same sample position with areas of 0.471 mm $\times$ 0.471 mm (512 $\times$ 512 pixels). The laser transmission though the sample is also acquired as an image in a PMT positioned after the microscope condenser. The focus was selected by optimizing the SHG intensity and then three images were collected, one at the focal position and one at each focal planes at 1 $\upmu$m above and below the optimal position. The acquisition of the three images allowed to assure the best focal position and they were also used by the software to eliminate noise. The scanning time for each image is 2.71 s. For the samples with a weak SHG intensity up to 5 images were accumulated, and the overall acquisition time was about 60 s.

We have acquired images for 836 representative areas of the NMT and the other histological types in the sections stained with H&E. On the histological slides in which normal mammary regions were selected the images were acquired for the 10 most representative areas of normal tissue. For the BMT slides, 5 peri-tumour areas and 5 intra-tumour areas were selected for measurements. The peri-tumour regions are defined from the fibrous tissue at the edges of the tumour, in the transition to the unchanged tissue; the intra-tumour regions correspond to areas with collagen within groups of cells with neoplastic growth. In the remaining cases, 10–15 most representative intra-tumour regions were selected for the measurements. The images from these selected areas were collected for analysis. The bright field microscopy images of the H&E stained tissue were obtained at the same areas for comparison.

### Image analysis

Quantitative analysis of the parameters for the collagen fibres and cellular segments were obtained using an open source software package named PyFibre (Python Fibrous Image Analysis Toolkit) that was developed to perform an automated image segmentation into collagen and cellular regions and to extract their parameters (available free on GitHub^47^). In the analysis procedure, the SHG and the TPEF signals, as well as a copy of the laser transmission data are used to better identify the collagen fibres and cellular features in the images. The SHG signal allows to identify the collagen fibres. For mapping out the locations of collagen fibres as a network we use a modified version of the FIbeR Extraction (FIRE) algorithm^61,62^. Additional information from the TPEF and transmission signals is then used to further refine the boundary between fibrous and cellular areas. The PyFibre analysis generates a database with all the metrics extracted from the images for the analysis, details of which can be found in the online software documentation^47^. The image segmentation allows the calculations to be performed specifically for the cellular and collagen fibre regions in the image. The values of the metrics evaluated are used as comparison parameters for normal tissues and the neoplastic tissue. The measurements of the SHG anisotropy characterize the organization of collagen fibres as purely isotropic regions (value 0) and regions in which the collagen fibres are perfectly aligned, or anisotropic (value 1). The extracted collagen fibre network allows to measure also the number of fibres and the fibre length. The cellular segment linearity is a measurement of the shape of the extracted cellular segments. It is proportional to the ratio of a circumference of a circle with the same area as the segment to the perimeter of cell segment. The value is considered as 0 if the cellular segment is circular and 1 if the segment is completely elongated. The fibre coverage area and cellular segment coverage area parameters are the image percentage covered by the fibre and cellular features, respectively. A strong negative correlation between the fibre segment and cellular segment coverage parameters is perhaps to be expected, but it is not guaranteed by the analysis method.

### Immunohistochemistry

Histological sections of 4 $\upmu$m thickness were prepared for immunohistochemistry reactions. A commercial anti-mouse/anti-rabbit detection kit (Novolink Polymer Detection System, Leica Biosystems, Newcastle Upon Tyne, United Kingdom) was used according to the manufacturer’s instructions. Antigenic recovery of estrogen receptor (ER), progesterone (PR), Ki67 and HER2 were performed in steam heat (Pascal) with pH 6.0 citrate (Dako Cytomation Target Retrieval Solution, Dako, Glostrup, Denmark). The slides with the histological sections were incubated with the appropriate primary antibody for 16 hours in a humid chamber at 4 $^\circ$C, ER (1:50, clone 1D5, Dako), PR (1:50, hPRa2 clone, Neomarkers, Fremont, CA, USA), HER2 (1: 200, polyclonal, Dako) and Ki67 (1:50, MIB-1 clone, Dako). Immunoreactivity was visualized with the 3$^{\prime }$-diaminobenzidine chromogen (DAB Substrate System, Dako, Carpinteria, CA, USA) and contrasted with Mayer’s hematoxylin. Samples of breast tissue fragments from women positive for ER, PR, HER-2 and Ki67 were used as positive controls of the reactions. For negative controls, the primary antibody was replaced with phosphate buffered saline (PBS). The analysis of the slides, quantification of immunoreactions and the classification of immunophenotypes were performed according to Nunes et al.^63^. The antibodies are the standard ones used in our laboratory routine procedures and the antigenic specificity has already been tested according to works published by our group^55,64,65^ and works published by other groups^66,67^.

### Statistical analysis

One-way analysis of variance (ANOVA) and multiple comparisons using the Tukey’s HSD (honestly significant difference) test were used to compare means between the diagnostic groups and $p <0.05$ was considered statistically significant. The *p* values less than 0.001 are denoted by (***), *p* less than 0.01 are denoted by (**) and *p* less than (0.05) are denoted by (*).

For the correlation analysis between the evaluated variables, the Spearman Rank Correlation Coefficient statistical test and linear regression were used. For all analyses, a value of $p < 0.05$ was considered statistically significant. These analyses were performed using the Microsoft Windows software, Prism (version 7.0, GraphPad, San Diego, CA, United States). The prognostic value of the different clinical-pathological variables was determined in patients treated with surgery only, based on an initial univariate analysis to assess the cancer-specific survival rate according to the immunophenotype. The cancer-specific survival rate was estimated using the Kaplan–Meier curve and the comparisons between groups were performed using the Mantel–Cox log rank test. Then, a multivariate analysis was performed using the Cox regression model to estimate the risk ratio considering the variables with $p <0.05$ in the univariate analysis. In Cox’s final model, only variables with $p <0.05$ were maintained in the model. The multivariate analysis was performed using the Stata software version 14.0 (SatataCorp, College Station, TX, USA).
